# An information integration theory of consciousness

**DOI:** 10.1186/1471-2202-5-42

**Published:** 2004-11-02

**Authors:** Giulio Tononi

**Affiliations:** 1 Department of Psychiatry, University of Wisconsin, Madison, USA

## Abstract

**Background:**

Consciousness poses two main problems. The first is understanding the conditions that determine to what extent a system has conscious experience. For instance, why is our consciousness generated by certain parts of our brain, such as the thalamocortical system, and not by other parts, such as the cerebellum? And why are we conscious during wakefulness and much less so during dreamless sleep? The second problem is understanding the conditions that determine what kind of consciousness a system has. For example, why do specific parts of the brain contribute specific qualities to our conscious experience, such as vision and audition?

**Presentation of the hypothesis:**

This paper presents a theory about what consciousness is and how it can be measured. According to the theory, consciousness corresponds to the capacity of a system to integrate information. This claim is motivated by two key phenomenological properties of consciousness: differentiation – the availability of a very large number of conscious experiences; and integration – the unity of each such experience. The theory states that the quantity of consciousness available to a system can be measured as the Φ value of a complex of elements. Φ is the amount of causally effective information that can be integrated across the informational weakest link of a subset of elements. A complex is a subset of elements with Φ>0 that is not part of a subset of higher Φ. The theory also claims that the quality of consciousness is determined by the informational relationships among the elements of a complex, which are specified by the values of effective information among them. Finally, each particular conscious experience is specified by the value, at any given time, of the variables mediating informational interactions among the elements of a complex.

**Testing the hypothesis:**

The information integration theory accounts, in a principled manner, for several neurobiological observations concerning consciousness. As shown here, these include the association of consciousness with certain neural systems rather than with others; the fact that neural processes underlying consciousness can influence or be influenced by neural processes that remain unconscious; the reduction of consciousness during dreamless sleep and generalized seizures; and the time requirements on neural interactions that support consciousness.

**Implications of the hypothesis:**

The theory entails that consciousness is a fundamental quantity, that it is graded, that it is present in infants and animals, and that it should be possible to build conscious artifacts.

## Background

Consciousness is everything we experience. Think of it as what abandons us every night when we fall into dreamless sleep and returns the next morning when we wake up [1]. Without consciousness, as far as we are concerned, there would be neither an external world nor our own selves: there would be nothing at all. To understand consciousness, two main problems need to be addressed. [2,3]. The *first problem *is to understand the conditions that determine to what extent a system has consciousness. For example, why is it that certain parts of the brain are important for conscious experience, whereas others, equally rich in neurons and connections, are not? And why are we conscious during wakefulness or dreaming sleep, but much less so during dreamless sleep, even if the brain remains highly active? The *second problem *is to understand the conditions that determine what kind of consciousness a system has. For example, what determines the specific and seemingly irreducible quality of the different modalities (e.g. vision, audition, pain), submodalities (e.g. visual color and motion), and dimensions (e.g. blue and red) that characterize our conscious experience? Why do colors look the way they do, and different from the way music sounds, or pain feels? Solving the first problem means that we would know to what extent a physical system can generate consciousness – the *quantity *or level of consciousness. Solving the second problem means that we would know what kind of consciousness it generates – the *quality *or content of consciousness.

## Presentation of the hypothesis

### The first problem: What determines to what extent a system has conscious experience?

We all know that our own consciousness waxes when we awaken and wanes when we fall asleep. We may also know first-hand that we can "lose consciousness" after receiving a blow on the head, or after taking certain drugs, such as general anesthetics. Thus, everyday experience indicates that consciousness has a physical substrate, and that that physical substrate must be working in the proper way for us to be fully conscious. It also prompts us to ask, more generally, what may be the conditions that determine to what extent consciousness is present. For example, are newborn babies conscious, and to what extent? Are animals conscious? If so, are some animals more conscious than others? And can they feel pain? Can a conscious artifact be constructed with non-neural ingredients? Is a person with akinetic mutism – awake with eyes open, but mute, immobile, and nearly unresponsive – conscious or not? And how much consciousness is there during sleepwalking or psychomotor seizures? It would seem that, to address these questions and obtain a genuine understanding of consciousness, empirical studies must be complemented by a theoretical analysis.

#### Consciousness as information integration

The theory presented here claims that consciousness has to do with the capacity to integrate information. This claim may not seem self-evident, perhaps because, being endowed with consciousness for most of our existence, we take it for granted. To gain some perspective, it is useful to resort to some thought experiments that illustrate key properties of subjective experience: its informativeness, its unity, and its spatio-temporal scale.

##### Information

Consider the following thought experiment. You are facing a blank screen that is alternately on and off, and you have been instructed to say "light" when the screen turns on and "dark" when it turns off. A photodiode – a very simple light-sensitive device – has also been placed in front of the screen, and is set up to beep when the screen emits light and to stay silent when the screen does not. The first problem of consciousness boils down to this. When you differentiate between the screen being on or off, you have the conscious experience of "seeing" light or dark. The photodiode can also differentiate between the screen being on or off, but presumably it does not consciously "see" light and dark. What is the key difference between you and the photodiode that makes you "see" light consciously? (see Appendix, i)

According to the theory, the key difference between you and the photodiode has to do with how much *information *is generated when that differentiation is made. Information is classically defined as reduction of uncertainty among a number of alternatives outcomes when one of them occurs [4]. It can be measured by the entropy function, which is the weighted sum of the logarithm of the probability (p) of alternatives outcomes (i): H = - Σp_i_log_2_p_i_. Thus, tossing a fair coin and obtaining heads corresponds to 1 bit of information, because there are just two alternatives; throwing a fair die yields log_2_(6) ≈ 2.59 bits of information, because there are six equally likely alternatives (H decreases if some of the outcomes are more likely than others, as would be the case with a loaded die).

When the blank screen turns on, the photodiode enters one of its two possible alternative states and beeps. As with the coin, this corresponds to 1 bit of information. However, when you see the blank screen turn on, the state you enter, unlike the photodiode, is one out of an extraordinarily large number of possible states. That is, the photodiode's repertoire is minimally differentiated, while yours is immensely so. It is not difficult to see this. For example, imagine that, instead of turning homogeneously on, the screen were to display at random every frame from every movie that was or could ever be produced. Without any effort, each of these frames would cause you to enter a different state and "see" a different image. This means that when you enter the particular state ("seeing light") you rule out not just "dark", but an extraordinarily large number of alternative possibilities. Whether you think or not of the bewildering number of alternatives (and you typically don't), this corresponds to an extraordinary amount of information (see Appendix, ii). This point is so simple that its importance has been overlooked.

##### Integration

While the ability to differentiate among a very large number of states is a major difference between you and the lowly photodiode, by itself it is not enough to account for the presence of conscious experience. To see why, consider an idealized one megapixel digital camera, whose sensor chip is essentially a collection of one million photodiodes. Even if each photodiode in the sensor chip were just binary, the camera as such could differentiate among 2^1,000,000 ^states, an immense number, corresponding to 1,000,000 bits of information. Indeed, the camera would easily enter a different state for every frame from every movie that was or could ever be produced. Yet nobody would believe that the camera is conscious. What is the key difference between you and the camera?

According to the theory, the key difference between you and the camera has to do with *information integration*. From the perspective of an external observer, the camera chip can certainly enter a very large number of different states, as could easily be demonstrated by presenting it with all possible input signals. However, the sensor chip can be considered just as well as a collection of one million photodiodes with a repertoire of two states each, rather than as a single integrated system with a repertoire of 2^1,000,000 ^states. This is because, due to the absence of interactions among the photodiodes within the sensory chip, the state of each element is causally independent of that of the other elements, and no information can be integrated among them. Indeed, if the sensor chip were literally cut down into its individual photodiodes, the performance of the camera would not change at all.

By contrast, the repertoire of states available to you cannot be subdivided into the repertoire of states available to independent components. This is because, due to the multitude of causal interactions among the elements of your brain, the state of each element is causally dependent on that of other elements, which is why information can be integrated among them. Indeed, unlike disconnecting the photodiodes in a camera sensor, disconnecting the elements of your brain that underlie consciousness has disastrous effects. The integration of information in conscious experience is evident phenomenologically: when you consciously "see" a certain image, that image is experienced as an integrated whole and cannot be subdivided into component images that are experienced independently. For example, no matter how hard you try, for example, you cannot experience colors independent of shapes, or the left half of the visual field of view independently of the right half. And indeed, the only way to do so is to physically split the brain in two to prevent information integration between the two hemispheres. But then, such split-brain operations yield two separate subjects of conscious experience, each of them having a smaller repertoire of available states and more limited performance [5].

##### Spatio-temporal characteristics

Finally, it is important to appreciate that conscious experience unfolds at a characteristic spatio-temporal scale. For instance, it flows in time at a characteristic speed and cannot be much faster or much slower. No matter how hard you try, you cannot speed up experience to follow a move accelerated a hundred times, not can you slow it down if the movie has decelerated. Studies of how a percept is progressively specified and stabilized – a process called microgenesis – indicate that it takes up to 100–200 milliseconds to develop a fully formed sensory experience, and that the surfacing of a conscious thought may take even longer [6]. In fact, the emergence of a visual percept is somewhat similar to the developing of a photographic print: first there is just the awareness that something has changed, then that it is something visual rather than, say, auditory, later some elementary features become apparent, such as motion, localization, and rough size, then colors and shapes emerge, followed by the formation of a full object and its recognition – a sequence that clearly goes from less to more differentiated [6]. Other evidence indicates that a single conscious moment does not extend beyond 2–3 seconds [7]. While it is arguable whether conscious experience unfolds more akin to a series of discrete snapshots or to a continuous flow, its time scale is certainly comprised between these lower and upper limits. Thus, a phenomenological analysis indicates that consciousness has to do with the ability to integrate a large amount of information, and that such integration occurs at a characteristic spatio-temporal scale.

#### Measuring the capacity to integrate information: The Φ of a complex

If consciousness corresponds to the capacity to integrate information, then a physical system should be able to generate consciousness to the extent that it has a large repertoire of available states (information), yet it cannot be decomposed into a collection of causally independent subsystems (integration). How can one identify such an integrated system, and how can one measure its repertoire of available states [2,8]?

As was mentioned above, to measure the repertoire of states that are available to a system, one can use the entropy function, but this way of measuring information is completely insensitive to whether the information is integrated. Thus, measuring entropy would not allow us to distinguish between one million photodiodes with a repertoire of two states each, and a single integrated system with a repertoire of 2^1,000,000 ^states. To measure information integration, it is essential to know whether a set of elements constitute a causally integrated system, or they can be broken down into a number of independent or quasi-independent subsets among which no information can be integrated.

To see how one can achieve this goal, consider an extremely simplified system constituted of a set of elements. To make matters slightly more concrete, assume that we are dealing with a neural system. Each element could represent, for instance, a group of locally interconnected neurons that share inputs and outputs, such as a cortical minicolumn. Assume further that each element can go through discrete activity states, corresponding to different firing levels, each of which lasts for a few hundred milliseconds. Finally, for the present purposes, let us imagine that the system is disconnected from external inputs, just as the brain is virtually disconnected from the environment when it is dreaming.

##### Effective information

**Figure 1:**
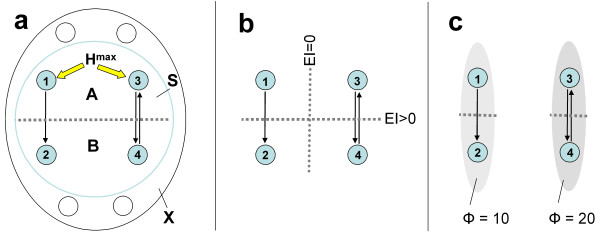
**Effective information, minimum information bipartition, and complexes. ***a. Effective information*. Shown is a single subset S of 4 elements ({1,2,3,4}, blue circle), forming part of a larger system X (black ellipse). This subset is bisected into A and B by a bipartition ({1,3}/{2,4}, indicated by the dotted grey line). Arrows indicate causally effective connections linking A to B and B to A across the bipartition (other connections may link both A and B to the rest of the system X). To measure EI(A→B), maximum entropy H^max ^is injected into the outgoing connections from A (corresponding to independent noise sources). The entropy of the states of B that is due to the input from A is then measured. Note that A can affect B directly through connections linking the two subsets, as well as indirectly via X. Applying maximum entropy to B allows one to measure EI(B→A). The effective information for this bipartition is EI(A 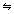 B) = EI(A→B) + EI(B→A). *b. Minimum information bipartition*. For subset S = {1,2,3,4}, the horizontal bipartition {1,3}/{2,4} yields a positive value of EI. However, the bipartition {1,2}/{3,4} yields EI = 0 and is a minimum information bipartition (MIB) for this subset. The other bipartitions of subset S = {1,2,3,4} are {1,4}/{2,3}, {1}/{2,3,4}, {2}/{1,3,4}, {3}/{1,2,4}, {4}/{1,2,3}, all with EI>0. *c. Analysis of complexes*. By considering all subsets of system X one can identify its complexes and rank them by the respective values of Φ – the value of EI for their minimum information bipartition. Assuming that other elements in X are disconnected, it is easy to see that Φ>0 for subset {3,4} and {1,2}, but Φ = 0 for subsets {1,3}, {1,4}, {2,3}, {2,4}, {1,2,3}, {1,2,4}, {1,3,4}, {2,3,4}, and {1,2,3,4}. Subsets {3,4} and {1,2} are not part of a larger subset having higher Φ, and therefore they constitute complexes. This is indicated schematically by having them encircled by a grey oval (darker grey indicates higher Φ). *Methodological note*. In order to identify complexes and their Φ(S) for systems with many different connection patterns, each system X was implemented as a stationary multidimensional Gaussian process such that values for effective information could be obtained analytically (details in [8]). Briefly, in order to identify complexes and their Φ(S) for systems with many different connection patterns, we implemented numerous model systems X composed of n neural elements with connections CON_ij _specified by a connection matrix CON(X) (no self-connections). In order to compare different architectures, CON(X) was normalized so that the absolute value of the sum of the afferent synaptic weights per element corresponded to a constant value w<1 (here w = 0.5). If the system's dynamics corresponds to a multivariate Gaussian random process, its covariance matrix COV(X) can be derived analytically. As in previous work, we consider the vector **X **of random variables that represents the activity of the elements of X, subject to independent Gaussian noise **R **of magnitude c. We have that, when the elements settle under stationary conditions, **X **= X * CON(X) + c**R**. By defining Q = (1-CON(X))^-1 ^and averaging over the states produced by successive values of **R**, we obtain the covariance matrix COV(X) = <X*X> = <Q^t ^* **R**^t ^* R * Q> = Q^t ^* Q, where the superscript t refers to the transpose. Under Gaussian assumptions, all deviations from independence among the two complementary parts A and B of a subset S of X are expressed by the covariances among the respective elements. Given these covariances, values for the individual entropies H(A) and H(B), as well as for the joint entropy of the subset H(S) = H(AB) can be obtained as, for example, H(A) = (1/2)ln [(2π e)^n^|COV(A)|], where |•| denotes the determinant. The mutual information between A and B is then given by MI(A;B) = H(A) + H(B) - H(AB). Note that MI(A:B) is symmetric and positive. To obtain the effective information between A and B within model systems, independent noise sources in A are enforced by setting to zero strength the connections within A and afferent to A. Then the covariance matrix for A is equal to the identity matrix (given independent Gaussian noise), and any statistical dependence between A and B must be due to the *causal *effects of A on B, mediated by the efferent connections of A. Moreover, all possible outputs from A that *could *affect B are evaluated. Under these conditions, EI(A→B) = MI(A^Hmax^;B). The independent Gaussian noise **R **applied to A is multiplied by c_p_, the perturbation coefficient, while the independent Gaussian noise applied to the rest of the system is given by c_i_, the intrinsic noise coefficient. Here c_p _= 1 and c_i _= 0.00001 in order to emphasize the role of the connectivity and minimize that of noise. To identify complexes and obtain their capacity for information integration, one considers every subset S of X composed of k elements, with k = 2,..., n. For each subset S, we consider all bipartitions and calculate EI(A 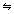 B) for each of them. We find the *minimum information bipartition *MIB(S), the bipartition for which the normalized effective information reaches a minimum, and the corresponding value of Φ(S). We then find the *complexes *of X as those subsets S with Φ>0 that are not included within a subset having higher Φ and rank them based on their Φ(S) value. The complex with the maximum value of Φ(S) is the *main complex*. MATLAB functions used for calculating effective information and complexes are at .

Consider now a subset S of elements taken from such a system, and the diagram of causal interactions among them (Fig. 1a). We want to measure the information generated when S enters a particular state out of its repertoire, but only to the extent that such information can be integrated, i.e. each state results from causal interactions within the system. How can one do so? One way is to divide S into two complementary parts A and B, and evaluate the responses of B that can be caused by all possible inputs originating from A. In neural terms, we try out all possible combinations of firing patterns as outputs from A, and establish how differentiated is the repertoire of firing patterns they produce in B. In information-theoretical terms, we give maximum entropy to the outputs from A (A^Hmax^), i.e. we substitute its elements with independent noise sources, and we determine the entropy of the responses of B that can be induced by inputs from A. Specifically, we define the *effective information *between A and B as EI(A→B) = MI(A^Hmax^;B). Here MI(A;B) = H(A) + H(B) - H(AB) stands for mutual information, a measure of the entropy or information shared between a source (A) and a target (B). Note that since A is substituted by independent noise sources, there are no causal effects of B on A; therefore the entropy shared by B and A is necessarily due to causal effects of A on B. Moreover, EI(A→B) measures all possible effects of A on B, not just those that are observed if the system were left to itself. Also, EI(A→B) and EI(B→A) in general are not symmetric. Finally, note that the value of EI(A→B) is bounded by A^Hmax ^and B^Hmax^, whichever is less. In summary, to measure EI(B→A), one needs to apply maximum entropy to the outputs from B, and determine the entropy of the responses of B that are induced by inputs from A. It should be apparent from the definition that EI(A→B) will be high if the connections between A and B are strong and specialized, such that different outputs from A will induce different firing patterns in B. On the other hand, EI(A→B) will be low or zero if the connections between A and B are such that different outputs from A produce scarce effects, or if the effect is always the same. For a given bipartition of a subset, then, the sum of the effective information for both directions is indicated as EI(A 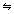 B) = EI(A→B) + EI(B→A). Thus, EI(A 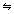 B) measures the repertoire of possible causal effects of A on B and of B on A.

##### Information integration

Based on the notion of effective information for a bipartition, we can assess how much information can be integrated within a system of elements. To this end, we note that a subset S of elements cannot integrate any information (as a subset) if there is a way to partition S in two parts A and B such that EI(A 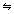 B) = 0 (Fig. 1b, vertical bipartition). In such a case, in fact, we would clearly be dealing with at least two causally independent subsets, rather than with a single, integrated subset. This is exactly what would happen with the photodiodes making up the sensor of a digital camera: perturbing the state of some of the photodiodes would make no difference to the state of the others. Similarly, a subset can integrate little information if there is a way to partition it in two parts A and B such that EI(A 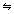 B) is low: the effective information across that bipartition is the limiting factor on the subset's information integration capacity. Therefore in order to measure the information integration capacity of a subset S, we should search for the bipartition(s) of S for which EI(A 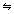 B) reaches a minimum (the informational "weakest link")." Since EI(A 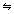 B) is necessarily bounded by the maximum entropy available to A or B, min{EI(A 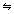 B)}, to be comparable over bipartitions, should be normalized by H^max^(A 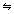 B) = min{H^max^(A); H^max^(B)}, the maximum information capacity for each bipartition. The *minimum information bipartition *^MIB^A 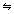 B of subset S – its 'weakest link' – is its bipartition for which the normalized effective information reaches a minimum, corresponding to min{EI(A 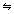 B)/H^max^(A 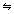 B)}. The *information integration *for subset S, or Φ(S), is simply the (non-normalized) value of EI(A 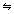 B) for the minimum information bipartition: Φ(S) = EI(^MIB^A 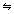 B). The symbol Φ is meant to indicate that the information (the vertical bar "I") is integrated within a single entity (the circle "O", see Appendix, iii).

##### Complexes

We are now in a position to establish which subsets are actually capable of integrating information, and how much of it (Fig. 1c). To do so, we consider every possible subset S of m elements out of the n elements of a system, starting with subsets of two elements (m = 2) and ending with a subset corresponding to the entire system (m = n). For each of them, we measure the value of Φ, and rank them from highest to lowest. Finally, we discard all those subsets that are included in larger subsets having higher Φ (since they are merely parts of a larger whole). What we are left with are *complexes *– individual entities that can integrate information. Specifically, a *complex *is a subset S having Φ>0 that is not included within a larger subset having higher Φ. For a complex, and only for a complex, it is appropriate to say that, when it enters a particular state out if its repertoire, it generates and amount of integrated information corresponding to its Φ value. Of the complexes that make up a given system, the one with the maximum value of Φ(S) is called the *main complex *(the maximum is taken over all combinations of m>1 out of n elements of the system). Some properties of complexes worth pointing out are, for instance, that a complex can be causally connected to elements that are not part of it (the input and output elements of a complex are called *ports-in *and *ports-out*, respectively). Also, the same element can belong to more than one complex, and complexes can overlap.

In summary, a system can be analyzed to identify its complexes – those subsets of elements that can integrate information, and each complex will have an associated value of Φ – the amount of information it can integrate (see Appendix, iv). To the extent that consciousness corresponds to the capacity to integrate information, complexes are the "subjects" of experience, being the locus where information can be integrated. Since information can only be integrated *within *a complex and not outside its boundaries, consciousness as information integration is necessarily subjective, private, and related to a single point of view or perspective [1,9]. It follows that elements that are part of a complex contribute to its conscious experience, while elements that are not part of it do not, even though they may be connected to it and exchange information with it through ports-in and ports-out.

##### Information integration over space and time

The Φ value of a complex is dependent on both spatial and temporal scales that determine what counts as a state of the underlying system. In general, there will be a "grain size", in both space and time, at which Φ reaches a maximum. In the brain, for example, synchronous firing of heavily interconnected groups of neurons sharing inputs and outputs, such as cortical minicolumns, may produce significant effects in the rest of the brain, while asynchronous firing of various combinations of individual neurons may be less effective. Thus, Φ values may be higher when considering as elements cortical minicolumns rather than individual neurons, even if their number is lower. On the other hand, Φ values would be extremely low with elements the size of brain areas. Time wise, Φ values in the brain are likely to show a maximum between tens and hundreds of milliseconds. It is clear, for example, that if one were to stimulate one half of the brain by inducing many different firing patterns, and examine what effects this produces on the other half, no stimulation pattern would produce any effect whatsoever after just a tenth of a millisecond, and Φ would be equal to zero. After say 100 milliseconds, however, there is enough time for differential effects to be manifested, and Φ would grow. On the other hand, given the duration of conduction delays and of postsynaptic currents, much longer intervals are not going to increase Φ values. Indeed, a neural system will soon settle down into states that become progressively more independent of the stimulation. Thus, the search for complexes of maximum Φ should occur over subsets at critical spatial and temporal scales.

To recapitulate, the theory claims that consciousness corresponds to the capacity to integrate information. This capacity, corresponding to the *quantity *of consciousness, is given by the Φ value of a complex. Φ is the amount of effective information that can be exchanged across the minimum information bipartition of a complex. A complex is a subset of elements with Φ>0 and with no inclusive subset of higher Φ. The spatial and temporal scales defining the elements of a complex and the time course of their interactions are those that jointly maximize Φ.

### The second problem: What determines the kind of consciousness a system has?

Even if we were reasonably sure that a system is conscious, it is not immediately obvious what kind of consciousness it would have. As was mentioned early on, our own consciousness comes in specific and seemingly irreducible qualities, exemplified by different modalities (e.g. vision, audition, pain), submodalities (e.g. visual color and motion), and dimensions (e.g. blue and red). What determines that colors look the way they do, and different from the way music sounds, or pain feels? And why can we not even imagine what a "sixth" sense would feel like? Or consider the conscious experience of others. Does a gifted musician experience the sound of an orchestra the same way you do, or is his experience richer? And what about bats [10]? Assuming that they are conscious, how do they experience the world they sense through echolocation? Is their experience of the world vision-like, audition-like, or completely alien to us? Unless we accept that the kind of consciousness a system has is arbitrary, there must be some necessary and sufficient conditions that determine exactly what kind of experiences it can have. This is the second problem of consciousness.

While it may not be obvious how best to address this problem, we do know that, just as the quantity of our consciousness depends on the proper functioning of a physical substrate – the brain, so does the quality of consciousness. Consider for example the acquisition of new discriminatory abilities, such as becoming expert at wine tasting. Careful studies have shown that we do not learn to distinguish among a large number of different wines merely by attaching the appropriate labels to different sensations that we had had all along. Rather, it seems that we actually enlarge and refine the set of sensations triggered by tasting wines. Similar observations have been made by people who, for professional reasons, learn to discriminate among perfumes, colors, sounds, tactile sensations, and so on. Or consider perceptual learning during development. While infants experience more than just a "buzzing confusion", there is no doubt that perceptual abilities undergo considerable refinement – just consider what your favorite red wine must have tasted like when all you had experienced was milk and water.

These examples indicate that the quality and repertoire of our conscious experience can change as a result of learning. What matters here is that such perceptual learning depends upon specific changes in the physical substrate of our consciousness – notably a refinement and rearranging of connections patterns among neurons in appropriate parts of the thalamocortical system (e.g [11]). Further evidence for a strict association between the quality of conscious experience and brain organization comes from countless neurological studies. Thus, we know that damage to certain parts of the cerebral cortex forever eliminates our ability to perceive visual motion, while leaving the rest of our consciousness seemingly intact. By contrast, damage to other parts selectively eliminates our ability to perceive colors. [12]. There is obviously something about the organization of those cortical areas that makes them contribute different qualities – visual motion and color – to conscious experience. In this regard, it is especially important that the same cortical lesion that eliminates the ability to perceive color or motion also eliminates the ability to remember, imagine, and dream in color or motion. By contrast, lesions of the retina, while making us blind, do not prevent us from remembering, imagining, and dreaming in color (unless they are congenital). Thus, it is something having to do with the organization of certain cortical areas – and not with their inputs from the sensory periphery – that determines the quality of conscious experiences we *can *have. What is this something?

#### Characterizing the quality of consciousness as a space of informational relationships: The effective information matrix

According to the theory, just as the quantity of consciousness associated with a complex is determined by the amount of information that can be integrated among its elements, the quality of its consciousness is determined by the informational relationships that causally link its elements [13]. That is, the way information can be integrated within a complex determines not only how much consciousness is has, but also what kind of consciousness. More precisely, the theory claims that the elements of a complex constitute the dimensions of an abstract relational space, the *qualia space*. The values of effective information among the elements of a complex, by defining the relationships among these dimensions, specify the structure of this space (in a simplified, Cartesian analogue, each element is a Cartesian axis, and the effective information values between elements define the angles between the axes, see Appendix, v). This relational space is sufficient to specify the quality of conscious experience. Thus, the reason why certain cortical areas contribute to conscious experience of color and other parts to that of visual motion has to do with differences in the informational relationships both *within *each area and *between *each area and the rest of the main complex. By contrast, the informational relationships that exist outside the main complex – including those involving sensory afferents – do not contribute either to the quantity or to the quality of consciousness.

**Figure 2:**
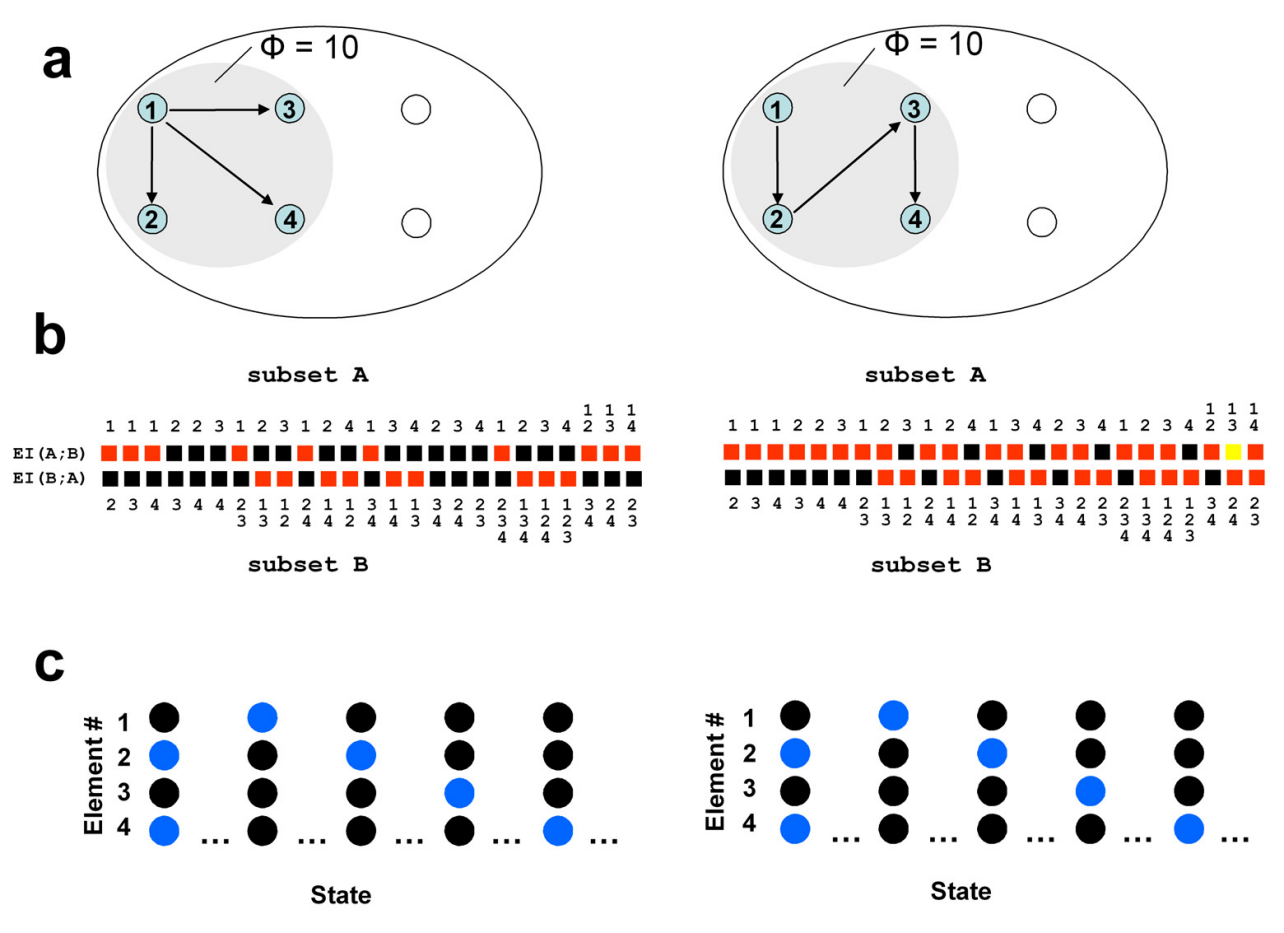
**Effective information matrix and activity states for two complexes having the same value of Φ. ***a. Causal interactions diagram and analysis of complexes*. Shown are two systems, one with a "divergent" architecture (left) and one with a "chain" architecture (right). The analysis of complexes shows that both contain a complex of four elements having a Φ value of 10. *b. Effective information matrix*. Shown is the effective information matrix for the two complexes above. For each complex, all bipartitions are indicated by listing one part (subset A) on the upper row and the complementary part (subset B) on the lower row. In between are the values of effective information from A to B and from B to A for each bipartition, color-coded as black (zero), red (intermediate value) and yellow (high value). Note that the effective information matrix is different for the two complexes, even though Φ is the same. The effective information matrix defines the set of informational relationships, or "qualia space" for each complex. Note that the effective information matrix refers exclusively to the informational relationships within the main complex (relationships with elements outside the main complex, represented here by empty circles, do not contribute to qualia space). *c. State diagram*. Shown are five representative states for the two complexes. Each is represented by the activity state of the four elements of each complex arranged in a column (blue: active elements; black: inactive ones). The five states can be thought of, for instance, as evolving in time due the intrinsic dynamics of the system or to inputs from the environment. Although the states are identical for the two complexes, their meaning is different because of the difference in the effective information matrix. The last four columns represent four special states, those corresponding to the activation of one element at a time. Such states, if achievable, would correspond most closely to the specific "quale" contributed by that particular element in that particular complex.

To exemplify, consider two very simple linear systems of four elements each (Fig. 2). Fig. 2a shows the diagram of causal interactions for the two systems. The system on the left is organized as a divergent digraph: element number 1 sends connections of equal strength to the other three elements. The analysis of complexes shows that this system forms a single complex having a Φ value of 10 bits. The system on the right is organized as a chain: element number 1 is connected to 2, which is connected to 3, which is connected to 4. This system also constitutes a single complex having a Φ value of 10 bits. Fig. 2b shows the effective information matrix for both complexes. This contains the values of EI between each subset of elements and every other subset, corresponding to all informational relationships among the elements (the first row shows the values in one direction, the second row in the reciprocal direction). The elements themselves define the dimensions of the qualia space of each complex, in this case four. The effective information matrix defines the relational structure of the space. This can be thought of as a kind of topology, in that the entries in the matrix can be considered to represent how close such dimensions are to each other (see Appendix, vi). It is apparent that, despite the identical value of Φ and the same number of dimensions, the informational relationships that define the space are different for the two complexes. For example, the divergent complex has many more zero entries, while the chain complex has one entry (subset {1 3} to subset {2 4}) that is twice as strong as all other non-zero entries.

These two examples are purely meant to illustrate how the space of informational relationships within a complex can be captured by the effective information matrix, and how that space can differ for two complexes having similar amounts of Φ and the same number of dimensions. Of course, for a complex having high values of Φ, such as the one underlying our own consciousness, qualia space would be extraordinarily large and intricately structured. Nevertheless, it is a central claim of the theory that the structure of phenomenological relationships should reflect directly that of informational relationships. For example, the conscious experiences of blue and red appear irreducible (red is not simply less of blue). They may therefore correspond to different dimensions of qualia space (different elements of the complex). We also know that, as different as blue and red may be subjectively, they are much closer to each other than they are, say, to the blaring of a trumpet. EI values between the neuronal groups underlying the respective dimensions should behave accordingly, being higher between visual elements than between visual and auditory elements. As to the specific quality of different modalities and submodalities, the theory predicts that they are due to differences in the set of informational relationships *within *the respective cortical areas and *between *each area and the rest of the main complex. For example, areas that are organized topographically and areas that are organized according to a "winner takes all" arrangement should contribute different kinds of experiences. Another prediction is that changes in the quality and repertoire of sensations as a result of perceptual learning would also correspond to a refinement of the informational relationships within and between the appropriate cortical areas belonging to the main complex. By contrast, the theory predicts that informational relationships outside a complex – including those among sensory afferents – should not contribute directly to the quality of conscious experience of that complex. Of course, sensory afferents, sensory organs, and ultimately the nature and statistics of external stimuli, play an essential role in shaping the informational relationships among the elements of the main complex – but such role is an indirect and historical one – played out through evolution, development, and learning [14] (see Appendix, vii).

#### Specifying each conscious experience: The state of the interaction variables

According to the theory, once the quantity and quality of conscious experience that a complex *can *have are specified, the particular conscious state or experience that the complex *will *have at any given time is specified by the activity state of its elements at that time (in a Cartesian analogue, if each element of the complex corresponds to an axis of qualia space, and effective information values between elements define the angles between the axes specifying the structure of the space, then the activity state of each element provides a coordinate along its axis, and each conscious state is defined by the set of all its coordinates). The relevant activity variables are those that mediate the informational relationships among the elements, that is, those that mediate effective information. For example, if the elements are local groups of neurons, then the relevant variables are their firing patterns over tens to hundreds of milliseconds.

The state of a complex at different times can be represented schematically by a state diagram as in Fig. 2c (for the divergent complex on the left and the chain complex on the right). Each column in the state diagram shows the activity values of all elements of a complex (here between 0 and 1). Different conscious states correspond to different patterns of activity distributed over *all *the elements of a complex, with no contribution from elements outside the complex. Each conscious state can thus be thought of as a different point in the multidimensional qualia space defined by the effective information matrix of a complex (see Appendix, viii). Therefore, a succession or flow of conscious states over time can be thought of as a trajectory of points in qualia space. The state diagram also illustrates some states that have particular significance (second to fifth column). These are the states with just one active element, and all other elements silent (or active at some baseline level). It is not clear whether such highly selective states can be achieved within a large neural complex of high Φ, such as that one that is postulated to underlie human consciousness. To the extent that this is possible, such highly selective states would represent the closest approximation to experiencing that element's specific contribution to consciousness – its quality or "quale". However, because of the differences in the qualia space between the two complexes, the same state over the four elements would correspond to different experiences (and mean different things) for the two complexes. It should also be emphasized that, in every case, it is the activity state of *all *elements of the complex that defines a given conscious state, and both active and inactive elements count.

To recapitulate, the theory claims that the quality of consciousness associated with a complex is determined by its effective information matrix. The effective information matrix specifies all informational relationships among the elements of a complex. The values of the variables mediating informational interactions among the elements of a complex specify the particular conscious experience at any given time.

## Testing the hypothesis

### Consciousness, information integration, and the brain

Based on a phenomenological analysis, we have argued that consciousness corresponds to the capacity to integrate information. We have then considered how such capacity can be measured, and we have developed a theoretical framework for consciousness as information integration. We will now consider several neuroanatomical or neurophysiological factors that are known to influence consciousness. After briefly discussing the empirical evidence, we will use simplified computer models to illustrate how these neuroanatomical and neurophysiological factors influence information integration. As we shall see, the information integration theory not only fits empirical observations reasonably well, but offers a principled explanation for them.

#### Consciousness is generated by a distributed thalamocortical network that is at once specialized and integrated

Ancient Greek philosophers disputed whether the seat of consciousness was in the lungs, in the heart, or in the brain. The brain's pre-eminence is now undisputed, and scientists are trying to establish which specific parts of the brain are important. For example, it is well established that the spinal cord is not essential for our conscious experience, as paraplegic individuals with high spinal transactions are fully conscious. Conversely, a well-functioning thalamocortical system is essential for consciousness [15]. Opinions differ, however, about the contribution of certain cortical areas [1,16-21]. Studies of comatose or vegetative patients indicate that a global loss of consciousness is usually caused by lesions that impair multiple sectors of the thalamocortical system, or at least their ability to work together as a system. [22-24]. By contrast, selective lesions of individual thalamocortical areas impair different submodalities of conscious experience, such as the perception of color or of faces [25]. Electrophysiological and imaging studies also indicate that neural activity that correlates with conscious experience is widely distributed over the cortex (e.g [20,26-29]). It would seem, therefore, that the neural substrate of consciousness is a distributed thalamocortical network, and that there is no single cortical area where it all comes together (see Appendix, ix).

The fact that consciousness as we know it is generated by the thalamocortical system fits well with the information integration theory, since what we know about its organization appears ideally suited to the integration of information. On the information side, the thalamocortical system comprises a large number of elements that are functionally specialized, becoming activated in different circumstances. [12,30]. Thus, the cerebral cortex is subdivided into systems dealing with different functions, such as vision, audition, motor control, planning, and many others. Each system in turn is subdivided into specialized areas, for example different visual areas are activated by shape, color, and motion. Within an area, different groups of neurons are further specialized, e.g. by responding to different directions of motion. On the integration side, the specialized elements of the thalamocortical system are linked by an extended network of intra- and inter-areal connections that permit rapid and effective interactions within and between areas [31-35]. In this way, thalamocortical neuronal groups are kept ready to respond, at multiple spatial and temporal scales, to activity changes in nearby and distant thalamocortical areas. As suggested by the regular finding of neurons showing multimodal responses that change depending on the context [36,37], the capacity of the thalamocortical system to integrate information is probably greatly enhanced by nonlinear switching mechanisms, such as gain modulation or synchronization, that can modify mappings between brain areas dynamically [34,38-40]. In summary, the thalamocortical system is organized in a way that appears to emphasize at once both functional specialization and functional integration.

**Figure 3:**
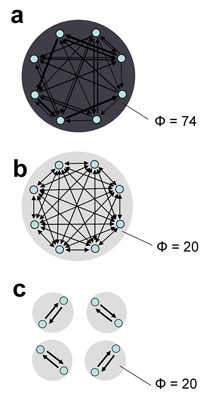
**Information integration for a thalamocortical-like architecture. ***a. Optimization of information integration for a system that is both functionally specialized and functionally integrated. *Shown is the causal interaction diagram for a network whose connection matrix was obtained by optimization for Φ (Φ = 74 bits). Note the heterogeneous arrangement of the incoming and outgoing connections: each element is connected to a different subset of elements, with different weights. Further analysis indicates that this network jointly maximizes functional specialization and functional integration among its 8 elements, thereby resembling the anatomical organization of the thalamocortical system [8]. *b. Reduction of information integration through loss of specialization*. The same amount of connectivity, distributed homogeneously to eliminate functional specialization, yields a complex with much lower values of Φ (Φ = 20 bits). *c. Reduction of information integration through loss of integration*. The same amount of connectivity, distributed in such a way as to form four independent modules to eliminate functional integration, yields four separate complexes with much lower values of Φ (Φ = 20 bits).

As shown by computer simulations, systems of neural elements whose connectivity jointly satisfies the requirements for functional specialization and for functional integration are well suited to integrating information. Fig. 3a shows a representative connection matrix obtained by optimizing for Φ starting from random connection weights. A graph-theoretical analysis indicates that connection matrices yielding the highest values of information integration (Φ = 74 bits) share two key characteristics [8]. First, connection patterns are different for different elements, ensuring functional specialization. Second, all elements can be reached from all other elements of the network, ensuring functional integration. Thus, simulated systems having maximum Φ appear to require both functional specialization and functional integration. In fact, if functional specialization is lost by replacing the heterogeneous connectivity with a homogeneous one, or if functional integration is lost by rearranging the connections to form small modules, the value of Φ decreases considerably (Fig 3b,3c). Further simulations show that it is possible to construct a large complex of high Φ by joining smaller complexes through reciprocal connections [8]. In the thalamocortical system, reciprocal connections linking topographically organized areas may be especially effective with respect to information integration. In summary, the coexistence of functional specialization and functional integration, epitomized by the thalamocortical system [30], is associated with high values of Φ.

#### Other brain regions with comparable numbers of neurons, such as the cerebellum, do not contribute to conscious experience

Consider now the cerebellum. This brain region contains more neurons than the cerebral cortex, has huge numbers of synapses, and receives mapped inputs from the environment and controls several outputs. However, in striking contrast to the thalamocortical system, lesions or ablations indicate that the direct contribution of the cerebellum to conscious experience is minimal. Why is this the case?

According to the theory, the reason lies with the organization of cerebellar connections, which is radically different from that of the thalamocortical system and is not well suited to information integration. Specifically, the organization of the connections is such that individual patches of cerebellar cortex tend to be activated independently of one another, with little interaction possible between distant patches [41,42]. This suggests that cerebellar connections may not be organized so as to generate a large complex of high Φ, but rather to give rise to many small complexes each with a low value of Φ. Such an organization seems to be highly suited for both the learning and the rapid, effortless execution of informationally insulated subroutines.

**Figure 4:**
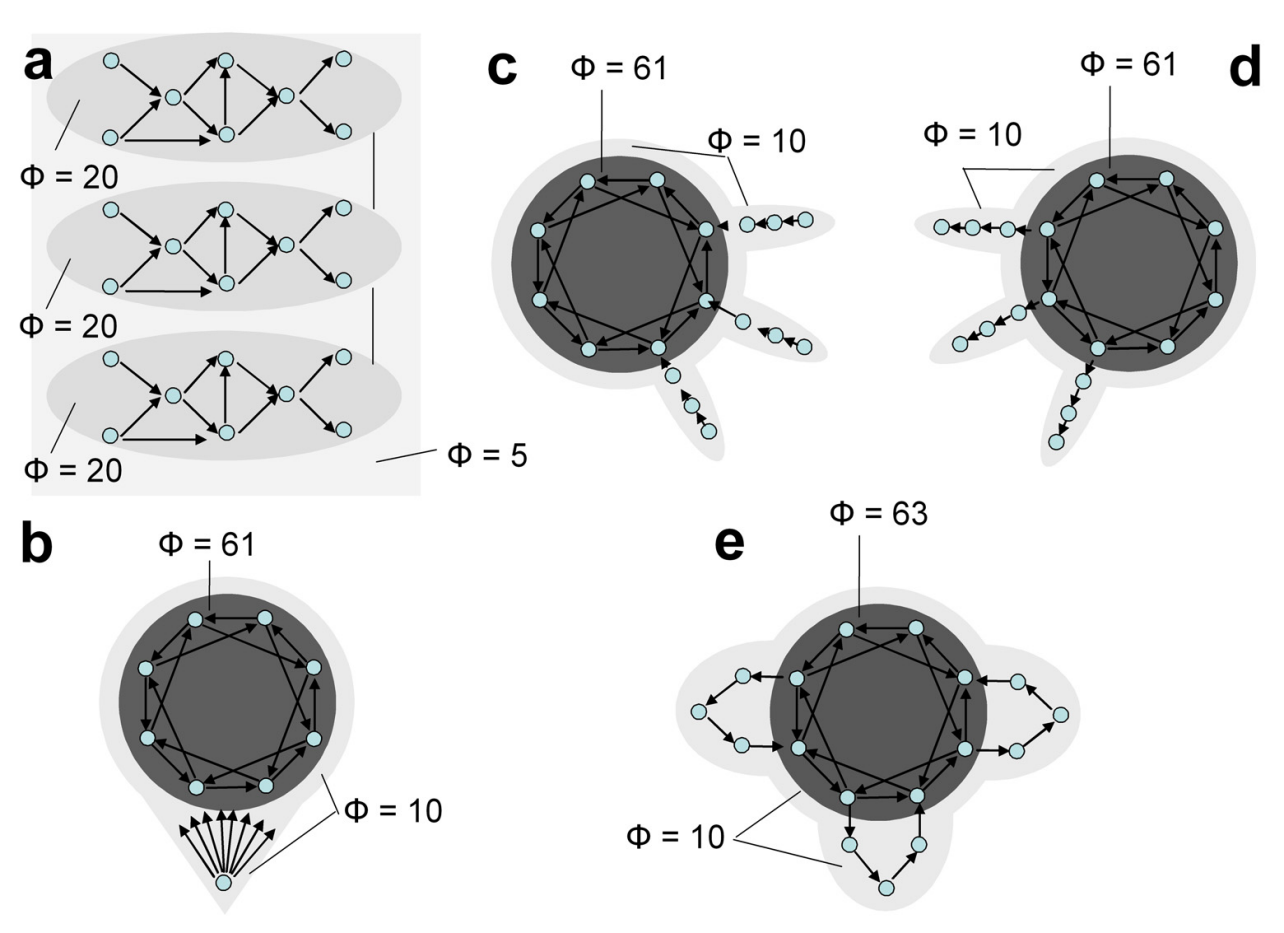
**Information integration and complexes for other neural-like architectures. ***a. Schematic of a cerebellum-like organization*. Shown are three modules of eight elements each, with many feed forward and lateral connections within each module but minimal connections among them. The analysis of complexes reveals three separate complexes with low values of Φ (Φ = 20 bits). There is also a large complex encompassing all the elements, but its Φ value is extremely low (Φ = 5 bits). *b. Schematic of the organization of a reticular activating system. *Shown is a single subcortical "reticular" element providing common input to the eight elements of a thalamocortical-like main complex (both specialized and integrated, Φ = 61 bits). Despite the diffuse projections from the reticular element on the main complex, the complex comprising all 9 elements has a much lower value of Φ (Φ = 10 bits). *c. Schematic of the organization of afferent pathways*. Shown are three short chains that stand for afferent pathways. Each chain connects to a port-in of a main complex having a high value of Φ (61 bits) that is thalamocortical-like (both specialized and integrated). Note that the afferent pathways and the elements of the main complex together constitute a large complex, but its Φ value is low (Φ = 10 bits). Thus, elements in afferent pathways can affect the main complex without belonging to it. *d. Schematic of the organization of efferent pathways*. Shown are three short chains that stand for efferent pathways. Each chain receives a connection from a port-out of the thalamocortical-like main complex. Also in this case, the efferent pathways and the elements of the main complex together constitute a large complex, but its Φ value is low (Φ = 10 bits). *e. Schematic of the organization of cortico-subcortico-cortical loops*. Shown are three short chains that stand for cortico-subcortico-cortical loops, which are connected to the main complex at both ports-in and ports-out. Again, the subcortical loops and the elements of the main complex together constitute a large complex, but its Φ value is low (Φ = 10 bits). Thus, elements in loops connected to the main complex can affect it without belonging to it. Note, however, that the addition of these three loops slightly increased the Φ value of the main complex (from Φ = 61 to Φ = 63 bits) by providing additional pathways for interactions among its elements.

This concept is illustrated in Fig. 4a, which shows a strongly *modular *network, consisting of three modules of eight strongly interconnected elements each. This network yields Φ = 20 bits for each of its three modules, which form the system's three complexes. This example indicates that, irrespective of how many elements and connections are present in a neural structure, if that structure is organized in a strongly modular manner with little interactions among modules, complex size and Φ values are necessarily low. According to the information integration theory, this is the reason why these systems, although computationally very sophisticated, contribute little to consciousness. It is also the reason why there is no conscious experience associated with hypothalamic and brainstem circuits that regulate important physiological variables, such as blood pressure.

#### Subcortical centers can control consciousness by modulating the readiness of the thalamocortical system without contributing directly to it

It has been known for a long time that lesions in the reticular formation of the brainstem can produce unconsciousness and coma. Conversely, stimulating the reticular formation can arouse a comatose animal and activate the thalamocortical system, making it ready to respond to stimuli [43]. Groups of neurons within the reticular formation are characterized by diffuse projections to many areas of the brain. Many such groups release neuromodulators such as acetylcholine, histamine, noradrenaline, serotonin, dopamine, and glutamate (acting on metabotropic receptors) and can have extremely widespread effects on both neural excitability and plasticity [44]. However, it would seem that the reticular formation, while necessary for the normal functioning of the thalamocortical system and therefore for the occurrence of conscious experience, may not contribute much in terms of specific dimensions of consciousness – it may work mostly like an external on-switch or as a transient booster of thalamocortical firing.

Such a role can be explained readily in terms of information integration. As shown in Fig. 4b, neural elements that have widespread and effective connections to a main complex of high Φ may nevertheless remain informationally excluded from it. Instead, they are part of a larger complex having a much lower value of Φ.

#### Neural activity in sensory afferents to the thalamocortical system can determine what we experience without contributing directly to it

What we see usually depends on the activity patterns that occur in the retina and that are relayed to the brain. However, many observations suggest that retinal activity does not contribute directly to conscious experience. Retinal cells surely can tell light from dark and convey that information to visual cortex, but their rapidly shifting firing patterns do not correspond well with what we perceive. For example, during blinks and eye movements retinal activity changes dramatically, but visual perception does not. The retina has a blind spot at the exit of the optic nerve where there are no photoreceptors, and it has low spatial resolution and no color sensitivity at the periphery of the visual field, but we are not aware of any of this. More importantly, lesioning the retina does not prevent conscious visual experiences. For example, a person who becomes retinally blind as an adult continues to have vivid visual images and dreams. Conversely, stimulating the retina during sleep by keeping the eyes open and presenting various visual inputs does not yield any visual experience and does not affect visual dreams. Why is it that retinal activity usually determines what we see through its action on thalamocortical circuits, but does not contribute directly to conscious experience?

As shown in Fig. 4c, adding or removing multiple, segregated incoming pathways does not change the composition of the main complex, and causes little change in its Φ. While the incoming pathways do participate in a larger complex together with the elements of the main complex, the Φ value of this larger complex is very low, being limited by the effective information between each afferent pathway and its port in at the main complex. Thus, input pathways providing powerful inputs to a complex add nothing to the information it integrates if their effects are entirely accounted for by ports-in.

#### Neural activity in motor efferents from the thalamocortical system, while producing varied behavioral outputs, does not contribute directly to conscious experience

In neurological practice, as well as in everyday life, we tend to associate consciousness with the presence of a diverse behavioral repertoire. For example, if we ask a lot of different questions and for each of them we obtain an appropriate answer, we generally infer that a person is conscious. Such a criterion is not unreasonable in terms of information integration, given that a wide behavioral repertoire is usually indicative of a large repertoire of internal states that are available to an integrated system. However, it appears that neural activity in motor pathways, which is necessary to bring about such diverse behavioral responses, does not in itself contribute to consciousness. For example, patients with the locked-in syndrome, who are completely paralyzed except for the ability to gaze upwards, are fully conscious. Similarly, while we are completely paralyzed during dreams, consciousness is not impaired by the absence of behavior. Even lesions of central motor areas do not impair consciousness.

Why is it that neurons in motor pathways, which can produce a large repertoire of different outputs and thereby relay a large amount of information about different conscious states, do not contribute directly to consciousness? As shown in Fig. 4d, adding or removing multiple, segregated outgoing pathways to a main complex does not change the composition of the main complex, and does not change its Φ value. Like incoming pathways, outgoing pathways do participate in a larger complex together with the elements of the main complex, but the Φ value of this larger complex is very low, being limited by the effective information between each port-out of the main complex and its effector targets.

#### Neural processes in cortico-subcortico-cortical loops, while important in the production and sequencing of action, thought, and language, do not contribute directly to conscious experience

Another set of neural structures that may not contribute directly to conscious experience are subcortical structures such as the basal ganglia. The basal ganglia are large nuclei that contain many circuits arranged in parallel, some implicated in motor and oculomotor control, others, such as the dorsolateral prefrontal circuit, in cognitive functions, and others, such as the lateral orbitofrontal and anterior cingulate circuits, in social behavior, motivation, and emotion [45]. Each basal ganglia circuit originates in layer V of the cortex, and through a last step in the thalamus, returns to the cortex, not far from where the circuit started [46]. Similarly arranged cortico-ponto-cerebello-thalamo-cortical loops also exist. Why is it that these complicated neural structures, which are tightly connected to the thalamocortical system at both ends, do not seem to provide much direct contribution to conscious experience? (see Appendix, x)

As shown in Fig. 4e, the addition of many parallel cycles also generally does not change the composition of the main complex, although Φ values can be altered (see Appendix, xi). Instead, the elements of the main complex and of the connected cycles form a joint complex that can only integrate the limited amount of information exchanged within each cycle. Thus, subcortical cycles or loops implement specialized subroutines that are capable of influencing the states of the main thalamocortical complex without joining it. Such informationally insulated cortico-subcortical loops could constitute the neural substrates for many unconscious processes that can affect and be affected by conscious experience [3,47]. It is likely that new informationally insulated loops can be created through learning and repetition. For example, when first performing a new task, we are conscious of every detail of it, we make mistakes, are slow, and must make an effort. When we have learned the task well, we perform it better, faster, and with less effort, but we are also less aware of it. As suggested by imaging results, a large number of neocortical regions are involved when we first perform a task. With practice, activation is reduced or shifts to different circuits [48]. According to the theory, during the early trials, performing the task involves many regions of the main complex, while later certain aspects of the task are delegated to neural circuits, including subcortical ones, that are informationally insulated.

#### Many neural processes within the thalamocortical system may also influence conscious experience without contributing directly to it

Even within the thalamocortical system proper, a substantial proportion of neural activity does not appear to contribute directly to conscious experience. For example, what we see and hear requires elaborate computational processes dealing with figure-ground segregation, depth perception, object recognition, and language parsing, many of which take place in the thalamocortical system. Yet we are not aware of all this diligent buzzing: we just *see *objects, separated from the background and laid out in space, and know what they are, or *hear *words, nicely separated from each other, and know what they mean. As an example, take binocular rivalry, where the two eyes view two different images, but we perceive consciously just one image at a time, alternating in sequence. Recordings in monkeys have shown that the activity of visual neurons in certain cortical areas, such as the inferotemporal cortex, follows faithfully what the subject perceives consciously. However, in other areas, such as primary visual cortex, there are many neurons that respond to the stimulus presented to the eye, whether or not the subject is perceiving it [49]. Neuromagnetic studies in humans have shown that neural activity correlated with a stimulus that is not being consciously perceived can be recorded in many cortical areas, including the front of the brain. [26]. Why does the firing of many cortical neurons carrying out the computational processes that enable object recognition (or language parsing) not correspond to anything conscious?

The situation is similar on the executive side of consciousness. When we plan to do or say something, we are vaguely conscious of what we intend, and presumably these intentions are reflected in specific firing patterns of certain neuronal groups. Our vague intentions are then translated almost miraculously into the right words, and strung together to form a syntactically correct sentence that conveys what we meant to say. And yet again, we are not at all conscious of the complicated processing that is needed to carry out our intentions, much of which takes place in the cortex. What determines whether the firing of neurons within the thalamocortical system contributes directly to consciousness or not? According to the information integration theory, the same considerations that apply to input and output circuits and to cortico-subcortico-cortical loops also apply to circuits and loops contained entirely within the thalamocortical system. Thus, the theory predicts that activity within certain cortical circuits does not contribute to consciousness because such circuits implement informationally insulated loops that remain outside of the main thalamocortical complex. At this stage, however, it is hard to say precisely which cortical circuits may be informationally insulated. Are primary sensory cortices organized like massive afferent pathways to a main complex "higher up" in the cortical hierarchy? Is much of prefrontal cortex organized like a massive efferent pathway? Do certain cortical areas, such as those belonging to the dorsal visual stream, remain partly segregated from the main complex? Do interactions *within *a cortico-thalamic minicolumn qualify as intrinsic mini-loops that support the main complex without being part of it? Unfortunately, answering these questions and properly testing the predictions of the theory requires a much better understanding of cortical neuroanatomy than is presently available [50,51].

#### Consciousness can be split if the thalamocortical system is split

Studies of split-brain patients, whose corpus callosum was sectioned for therapeutic reasons, show that each hemisphere has its own, private conscious experience. The dominant, linguistically competent hemisphere does not seem to suffer a major impairment of consciousness after the operation. The non-dominant hemisphere, although it loses some important abilities and its residual capacities are harder to assess, also appears to be conscious. [5]. Some information, e.g. emotional arousal, seems to be shared across the hemispheres, probably thanks to subcortical common inputs.

**Figure 5:**
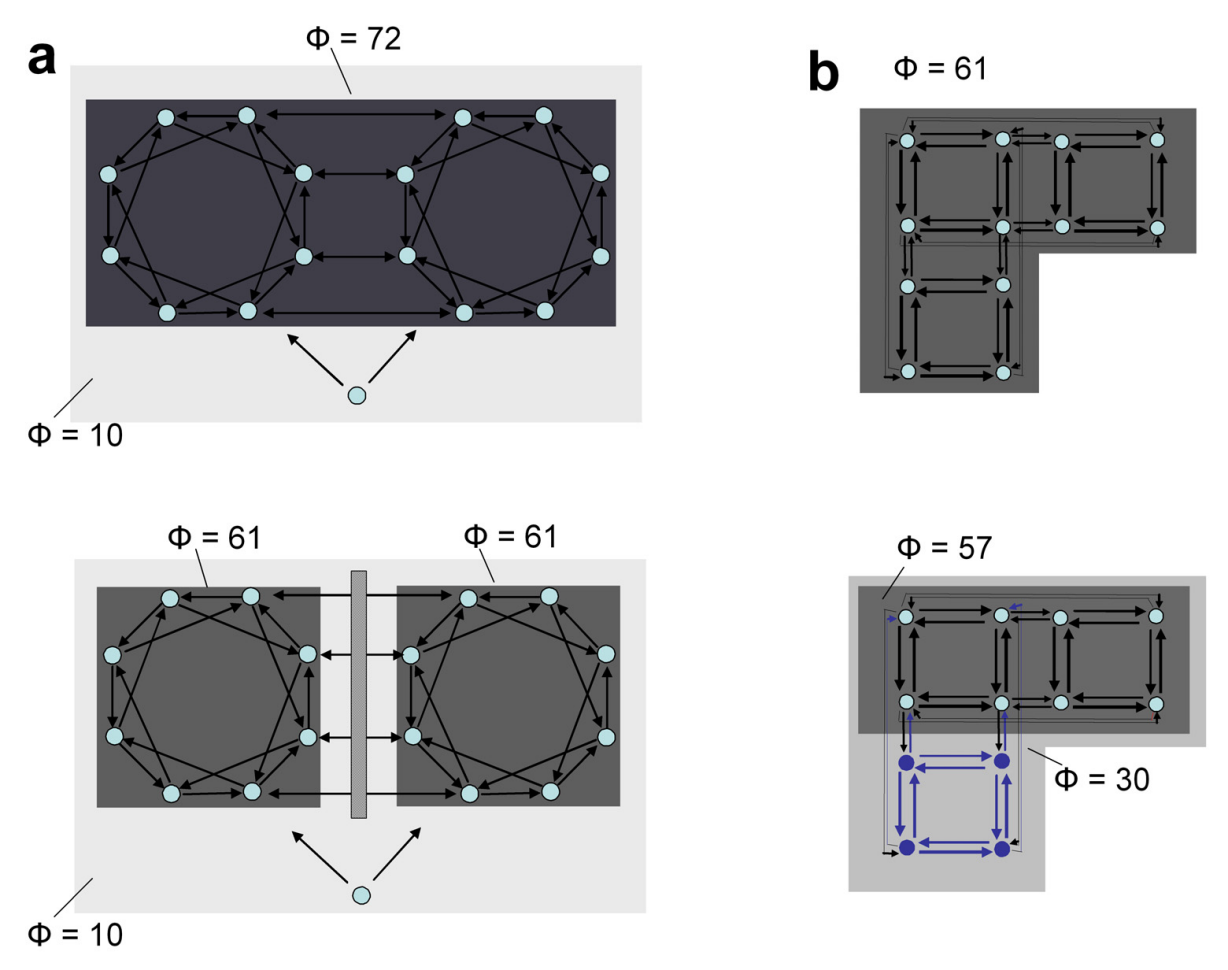
**Information integration and complexes after anatomical and functional disconnections. ***a. Schematic of a split-brain-like anatomical disconnection*. *Top*. Shown is a large main complex obtained by connecting two thalamocortical-like subsets through "callosum-like" reciprocal connections. There is also a single element that projects to all other elements, representing "subcortical" common input. Note that the Φ value for the main complex (16 elements) is high (Φ = 72 bits). There is also a larger complex including the "subcortical" element, but its Φ value is low (Φ = 10). *Bottom*. If the "callosum-like" connections are cut, one obtains two 8-element complexes, corresponding to the two "hemispheres", whose Φ value is reduced but still high (Φ = 61 bits). The two "hemispheres" still share some information due to common input from the "subcortical" element with which they form a large complex of low Φ. *b. Schematic of a functional disconnection. Top*. Shown is a large main complex obtained by linking with reciprocal connections a "supramodal" module of four elements (cornerstone) with a "visual" module (to its right) and an "auditory" module (below). Note that there are no direct connections between the "visual" and "auditory" modules. The 12 elements together form a main complex with Φ = 61 bits. *Bottom*. If the "auditory" module is functionally disconnected from the "supramodal" one by inactivating its four elements (indicated in blue), the main complex shrinks to include just the "supramodal" and "visual" modules. In this case, the Φ value is only minimally reduced (Φ = 57 bits).

Viewing consciousness as information integration suggests straightforward explanations for these puzzling observations. Consider the simplified model in Fig. 5a. A main complex having high Φ includes two sets of elements ("hemispheres") having similar internal architecture that are joined by "callosal" connections (top panel). When the callosal connections are cut (bottom panel), the single main complex splits and is replaced by two smaller complexes corresponding to the two hemispheres. There is also a complex, of much lower Φ, which includes both hemispheres and a "subcortical" element that provide them with common input. Thus, there is a sense in which the two hemispheres still form an integrated entity, but the information they share is minimal (see Appendix, xii).

#### Some parts of the thalamocortical system may contribute to conscious experience at one time and not at another

Until now, we have considered structural aspects of the organization of the nervous system that, according to the information integration theory, explain why certain parts of the brain contribute directly to consciousness and others do not, or much less so. In addition to neuroanatomical factors, neurophysiological factors are also important in determining to what extent a given neural structure can integrate information. For example, anatomical connections between brain regions may or may not be functional, depending on both pathological or physiological factors. Functional disconnections between certain parts of the brain and others are thought to play a role in psychiatric conversion and dissociative disorders, may occur during dreaming, and may be implicated in conditions such as hypnosis. Thus, functional disconnections, just like anatomical disconnections, may lead to a restriction of the neural substrate of consciousness.

It is also likely that certain attentional phenomena may correspond to changes in the neural substrate of consciousness. For example, when one is absorbed in thought, or focused exclusively on a given sensory modality, such as vision, the neural substrate of consciousness may not be the same as when we are diffusely monitoring the environment. Phenomena such as the attentional blink, where a fixed sensory input may at times make it to consciousness and at times not, may also be due to changes in functional connectivity: access to the main thalamocortical complex may be enabled or not based on dynamics intrinsic to the complex [52]. Phenomena such as binocular rivalry may also be related, at least in part, to dynamic changes in the composition of the main thalamocortical complex caused by transient changes in functional connectivity [53]. At present, however, it is still not easy to determine whether a particular group of neurons is excluded from the main complex because of hard-wired anatomical constraints, or is transiently disconnected due to functional changes.

Figure 5b (top panel) shows a simple model obtained by taking three subsets of elements of (relatively) high Φ and connecting them through reciprocal connections. Specifically, the first subset, which stands for supramodal areas of the brain, is reciprocally connected to the second and third subsets, which stand for visual and auditory areas, respectively. In this idealized example, the visual and auditory subsets are not connected directly among themselves. As one can see, the three subsets thus connected form a single main complex having a Φ value of 61 bits. In the bottom panel, the auditory subset has been disconnected, in a functional sense, by mimicking a profound deactivation of its elements. The result is that the main complex shrinks and the auditory subset ends up outside the main complex. Note, however, that in this particular case the value of Φ changes very little (57 bits), indicating that it might be possible for the borders of the main complex to change dynamically while the amount of consciousness is not substantially altered. What would change, of course, would be the configuration of the space of informational relationships. These simulations suggest that attentional mechanisms may work both by changing neuronal firing rates, and therefore saliency within qualia space, as well as by modifying neuronal readiness to fire, and therefore the boundaries of the main complex and of qualia space itself. This is why the set of elements underlying consciousness is not static, but can be considered to form a "*dynamic complex*" or "*dynamic core*" [1,9].

#### Depending on certain neurophysiological parameters, the same thalamocortical network can generate much or little conscious experience

Another example of the importance of neurophysiological parameters is provided by sleep – the most familiar of the alterations of consciousness, and yet one of the most striking. Upon awakening from dreamless sleep, we have the peculiar impression that for a while we were not there at all nor, as far as we are concerned, was the rest of the world. This everyday observation tells us vividly that consciousness can come and go, grow and shrink. Indeed, if we did not sleep, it might be hard to imagine that consciousness is not a given, but depends somehow on the way our brain is functioning. The loss of consciousness between falling asleep and waking up is relative, rather than absolute. [54]. Thus, careful studies of mental activity reported immediately after awakening have shown that some degree of consciousness is maintained during much of sleep. Many awakenings, especially from rapid eye movement (REM) sleep, yield dream reports, and dreams can be at times as vivid and intensely conscious as waking experiences. Dream-like consciousness also occurs during various phases of slow wave sleep, especially at sleep onset and during the last part of the night. Nevertheless, a certain proportion of awakenings do not yield any dream report, suggesting a marked reduction of consciousness. Such "empty" awakenings typically occur during the deepest stages of slow wave sleep (stages 3 and 4), especially during the first half of the night.

Which neurophysiological parameters are responsible for the remarkable changes in the quantity and quality of conscious experience that occur during sleep? We know for certain that the brain does not simply shut off during sleep. During REM sleep, for example, neural activity is as high, if not higher, than during wakefulness, and EEG recordings show low-voltage fast-activity. This EEG pattern is known as "activated" because cortical neurons, being steadily depolarized and close to their firing threshold, are ready to respond to incoming inputs. Given these similarities, it is perhaps not surprising that consciousness should be present during both states. Changes in the quality of consciousness, however, do occur, and they correspond closely to relative changes in the activation of different brain areas. [54].

During slow wave sleep, average firing rates of cortical neurons are also similar to those observed during quiet wakefulness. However, due to changes in the level of certain neuromodulators, virtually all cortical neurons engage in slow oscillations at around 1 Hz, which are reflected in slow waves in the EEG [55]. Slow oscillations consist of a depolarized phase, during which the membrane potential of cortical neurons is close to firing threshold and spontaneous firing rates are similar to quiet wakefulness, and of a hyperpolarized phase, during which neurons become silent and are further away from firing threshold. From the perspective of information integration, a reduction in the readiness to respond to stimuli during the hyperpolarization phase of the slow oscillation would imply a reduction of consciousness. It would be as if we were watching very short fragments of a movie interspersed with repeated unconscious "blanks" in which we cannot see, think, or remember anything, and therefore have little to report. A similar kind of unreadiness to respond, associated with profound hyperpolarization, is found in deep anesthesia, another condition where consciousness is impaired. Studies using transcranial magnetic stimulation in conjunction with high-density EEG are currently testing how response readiness changes during the sleep waking cycle.

From the perspective of information integration, a reduction of consciousness during certain phases of sleep would occur even if the brain remained capable of responding to perturbations, provided its response were to lack differentiation. This prediction is borne out by detailed computer models of a portion of the visual thalamocortical system (Hill and Tononi, in preparation). According to these simulations, in the waking mode different perturbations of the thalamocortical network yield specific responses. In the sleep mode, instead, the network becomes bistable: specific effects of different perturbations are quickly washed out and their propagation impeded: the whole network transitions into the depolarized or into the hyperpolarized phase of the slow oscillation – a stereotypic response that is observed irrespective of the particular perturbation (see Appendix, xiii). And of course, this bistability is also evident in the spontaneous behavior of the network: during each slow oscillation, cortical neurons are either all firing or all silent, with little freedom in between. In summary, these simulations indicate that, even if the anatomical connectivity of a complex stays the same, a change in key parameters governing the readiness of neurons to respond and the differentiation of their responses may alter radically the Φ value of the complex, with corresponding consequences on consciousness.

#### Conscious experience and time requirements

Consciousness not only requires a neural substrate with appropriate anatomical structure and appropriate physiological parameters: it also needs time. As was mentioned earlier, studies of how a percept is progressively specified and stabilized indicate that it takes up to 100–200 milliseconds to develop a fully formed sensory experience, and that the surfacing of a conscious thought may take even longer. Experiments in which the somatosensory areas of the cerebral cortex were stimulated directly indicate that low intensity stimuli must be sustained for up to 500 milliseconds to produce a conscious sensation [56]. Multi-unit recordings in the primary visual cortex of monkeys show that, after a stimulus is presented, the firing rate of many neurons increases irrespective of whether the animal reports seeing a figure or not. After 80–100 milliseconds, however, their discharge accurately predicts the conscious detection of the figure. Thus, the firing of the same cortical neurons may correlate with consciousness at certain times, but not at other times [57]. What determines when the firing of the same cortical neurons contributes to conscious experience and when it does not? And why may it take up to hundreds of milliseconds before a conscious experience is generated?

The theory predicts that the time requirements for the generation of conscious experience in the brain emerge directly from the time requirements for the build-up of effective interactions among the elements of the main complex. As was mentioned above, if one were to perturb half of the elements of the main complex for less than a millisecond, no perturbations would produce any effect on the other half within this time window, and Φ would be equal to zero. After say 100 milliseconds, however, there is enough time for differential effects to be manifested, and Φ should grow. This prediction is confirmed by results obtained using large-scale computer simulations of the thalamocortical system, where the time course of causal interactions and functional integration can be studied in detail [38,58,59], Hill and Tononi, unpublished results). For example, in a model including nine functionally segregated visual areas, the time it takes for functionally specialized neurons located in several different areas to interact constructively and produce a specific, correlated firing pattern is at least 80 milliseconds [38]. These correlated firing patterns last for several hundred milliseconds. After one or more seconds, however, the network settles into spontaneous activity states that are largely independent of previous perturbations. Thus, the characteristic time scale for maximally differentiated responses in thalamocortical networks appears to be comprised between a few tens of milliseconds and a few seconds at the most.

In summary, the time scale of neurophysiological interactions needed to integrate information among distant cortical regions appears to be consistent with that required by psychophysical observations (microgenesis), by stimulation experiments, and by recording experiments.

## Summary: seeing blue

The previous examples show that the information integration theory is consistent with several empirical observations concerning the neural substrate of consciousness. Moreover, they show that the theory can provide a principled account of why consciousness is associated with certain parts of the brain rather than with others, and with certain global modes of functioning more than with others. To recapitulate the main tenets of the theory, it may be useful to reconsider the initial thought experiment.

Imagine again that you are comfortably facing a blank screen that is alternately on and off. When the screen turns on, you see a homogenous blue field, indeed for the sake of the argument we assume that you are having a "pure" perception of blue, unencumbered by extraneous percepts or thoughts (perhaps as can be achieved in certain meditative states). As you have been instructed, you signal your perception of blue by pushing a button. Now consider an extremely simplified scenario of the neural events that might accompany your seeing blue. When the screen turns on, a volley of activity propagates through the visual afferent pathways, involving successive stages such as retinal short wavelength cones, blue-yellow opponents cells, color constant cells, and so on. Eventually, this volley of activity in the visual afferent pathways leads to the firing of some neuronal groups in color-selective areas of the temporal lobe that, on empirical grounds, are our best bet for the neural correlate of blue: i) their activity correlates well with your perception of blue whether you see, imagine, or dream blue, in a way that is as stable and as susceptible to illusions as your perception of blue; ii) their microstimulation leads to the perception of blue; and iii) their selective lesion makes you unable to perceive blue. Let us assume, then, that these neuronal groups quickly increase their firing, and within a few tens of milliseconds they reach and then maintain increased levels of firing (see Appendix, xiv). We also assume that, at the same time, neuronal groups in neighboring cortical areas go on firing at a baseline level, largely unaffected by the blue light. These include neuronal groups in other visual areas that are selective for shape or movement; neuronal groups in auditory area that are selective for tones; and many others. On the other hand, the volley of activity originating in the retina does not exhaust itself by generating sustained activity in the color areas of the temporal lobe. Part of the volley proceeds at great speed and activates efferent motor pathways, which cause you to push the signaling button. Another part activates cortico-subcortico-cortical loops in your prefrontal cortex and basal ganglia, which almost make you speak the word "blue" aloud. In the meantime, many other parts of the brain are buzzing along, unaffected by what is going on in the visual system: cerebellar circuits are actively stabilizing your posture and gaze, and hypothalamic-brainstem circuits are actively stabilizing your blood pressure. What components in this simplified neural scenario are essential for your conscious experience of blue, and why?

The information integration theory makes several claims that lead to associated predictions. A first claim is that the neural substrate of consciousness as we know it is a complex of high Φ that is capable of integrating a large amount of information – the main complex. Therefore, *whether *a group of neurons contributes directly to consciousness is a function of its belonging to the main complex or not. In this example, the theory would predict that blue-selective neurons in some high-level color area should be *inside *the main complex; on the other hand, blue-sensitive neurons in afferent visual pathways, neurons in efferent pathways mediating the button-pressing response, neurons in cortico-subcortico-cortical and intracortical loops mediating subvocalization of the word "blue", neurons in the cerebellum controlling posture and neurons in hypothalamic-brainstem circuits controlling blood pressure should be *outside*. This even though these neurons may be equally active when you see blue, and even though some of them may be connected to elements of the main complex. In principle, joint microstimulation and recording experiments, and to some extent an analysis of patterns of synchronization, could determine participation in the main complex and test this prediction. The theory also predicts that blue-selective neurons in the main complex contribute to the conscious experience of blue only if their activation is sufficiently strong or sustained that they can make a difference, in informational terms, to the rest of the complex. Additional predictions are that, if a group of neurons that is normally part of the main complex becomes informationally disconnected from it, as could occur through attentional effects or in certain phases of sleep, the same group of neurons, firing in exactly the same way, would not contribute to consciousness. Moreover, according to the theory, the other groups of neurons within the main complex are essential to our conscious experience of blue even if, as in this example, they are not activated. This is not difficult to see. Imagine that, starting from an intact main complex, we were to remove one element after another, except for the active, blue-selective one. If an inactive element contributing to "seeing red" were removed, blue would not be experienced as blue anymore, but as some less differentiated color, perhaps not unlike those experienced by certain dichromats. If further elements of the main complex were removed, including those contributing to shapes, to sounds, to thoughts and so forth, one would soon drop to such a low level of consciousness that "seeing blue" would become meaningless: the "feeling" (and meaning) of the quale "blue" would have been eroded down to nothing. Indeed, while the remaining neural circuits may still be able to discriminate blue from other colors, they would do so very much as a photodiode does (see Appendix, xv).

A second claim of the theory is that the quality of consciousness is determined by the informational relationships within the main complex. Therefore, *how *a group of neurons contributes to consciousness is a function of its informational relationships inside the complex and not outside of it. In this example, blue-selective neurons within the main complex have become blue-selective no doubt thanks to the inputs received from the appropriate afferent pathways, and ultimately because of some aspects of the statistics of the environment and the resulting plastic changes throughout the brain. However, the theory predicts that their present firing contributes the quale "blue" exclusively because of their informational relationships within the main complex. If connections *outside *the main complex were to be manipulated, including the afferent color pathways, the experience elicited by activating the blue-selective neurons within the complex would stay the same. Conversely, if the relationships *inside *the main complex were to change, as could be done by changing the pattern of connections within the color-selective area and with the rest of the complex, so would the conscious experience of blue. That is, activating the same neurons would produce a different conscious experience.

## Implications of the hypothesis

To conclude, it is worth mentioning some of the implications that derive from the information integration theory of consciousness. At the most general level, the theory has ontological implications. It takes its start from phenomenology and, by making a critical use of thought experiments, it argues that subjective experience is one and the same thing as a system's capacity to integrate information. In this view, experience, that is, information integration, is a fundamental quantity, just as mass, charge or energy are. It follows that any physical system has subjective experience to the extent that it is capable of integrating information, irrespective of what it is made of. Thus, an intriguing implication of the theory is that it should be possible to construct conscious artifacts by endowing them with a complex of high Φ. Moreover, it should be possible to design the quality of their conscious experience by appropriately structuring their effective information matrix.

It also follows that consciousness is not an all-or-none property, but it is graded: to varying degrees, it should exist in most natural (and artificial) systems. Because the conditions needed to build complexes of high Φ are apparently not easy to achieve, however, correspondingly high levels of experience are probably available to only a few kinds of systems, primarily complex brains containing the right type of architecture for maximizing functional specialization and integration. A related implication is that consciousness should also exist, to varying degrees, at multiple spatial and temporal scales. However, it is likely that, in most systems, there are privileged spatial and temporal scales at which information integration reaches a maximum.

Consciousness is characterized here as a disposition or *potentiality *– in this case as the potential differentiation of a system's responses to all possible perturbations, yet it is undeniably *actual*. Consider another thought experiment: you could be in a coma for days, awaken to consciousness for just one second, and revert to a coma. As long as your thalamocortical system can function well for that one second, you will be conscious. That is, a system does not have to explore its repertoire of states to be conscious, or to know how conscious it is supposed to be: what counts is only that the repertoire is potentially available. While this may sound strange, fundamental quantities associated with physical systems can also be characterized as dispositions or potentialities, yet have actual effects. For example, mass can be characterized as a potentiality – say the resistance that a body would offer to acceleration by a force – yet it exerts undeniable effects, such as attracting other masses. This too has intriguing implications. For example, because in this view consciousness corresponds to the potential of an integrated system to enter a large number of states by way of causal interactions within it, experience is present as long as such potential is present, whether or not the system's elements are activated. Thus, the theory predicts that a brain where no neurons were activated, but were kept ready to respond in a differentiated manner to different perturbations, would be conscious (perhaps that nothing was going on). Also, because consciousness is a property of a system, not of a state, the state the system is in only determines which particular experience becomes actual at any given time, and not whether experience is present. Thus, a brain where each neuron were microstimulated to fire as an exact replica of your brain, but where synaptic interactions had been blocked, would be unconscious.

The theory predicts that consciousness depends exclusively on the ability of a system to integrate information, whether or not it has a strong sense of self, language, emotion, a body, or is immersed in an environment, contrary to some common intuitions. This prediction is consistent with the preservation of consciousness during REM sleep, when both input and output signals from and to the body are markedly reduced. Transient inactivation of brain areas mediating the sense of self, language, and emotion could assess this prediction in a more cogent manner.

Nevertheless, the theory recognizes that these same factors are important historically because they favor the development of neural circuits forming a main complex of high Φ. For example, the ability of a system to integrate information grows as that system incorporates statistical regularities from its environment and learns [14]. In this sense, the emergence of consciousness in biological systems is predicated on a long evolutionary history, on individual development, and on experience-dependent change in neural connectivity. Indeed, the theory also suggests that consciousness provides an adaptive advantage and may have evolved precisely because it is identical with the ability to integrate a lot of information in a short period of time. If such information is about the environment, the implication is that, the more an animal is conscious, the larger the number of variables it can take into account jointly to guide its behavior.

Another implication of the theory is that the presence and extent of consciousness can be determined, in principle, also in cases in which we have no verbal report, such as infants or animals, or in neurological conditions such as coma and vegetative states, minimally conscious states, akinetic mutism, psychomotor seizures, and sleepwalking. In practice, of course, measuring Φ accurately in such systems will not be easy, but approximations and informed guesses are certainly conceivable.

At present, the validity of this theoretical framework and the plausibility of its implications rest on its ability to account, in a coherent manner, for some basic phenomenological observations and for some elementary but puzzling facts about the relationship between consciousness and the brain. Experimental developments, especially of ways to stimulate and record concurrently the activity of broad regions of the brain, should permit stringent tests of some of the theory's predictions. Equally important will be the development of realistic, large-scale models of the anatomical organization of the brain. These models should allow a more rigorous measurement of how the capacity to integrate information relates to different brain structures and certain neurophysiological parameters [38,50,59]. Finally, the theoretical framework presented here aims primarily at understanding the necessary and sufficient conditions that determine the quantity and quality of consciousness at the most general level. Further theoretical developments will be required to address several issues that are central to the study of consciousness in a biological and psychological context, such as the relationship of consciousness to memory and language, higher order aspects of consciousness [60,61], and its relationship to the self) [62]. Undoubtedly, a full understanding of how the brain generates human consciousness remains a formidable task. However, if experimental investigations can be complemented by a principled theoretical approach, it may not lay beyond the reach of science.

## Appendix

i. The problem can also be posed in neural terms. When we see light, certain neurons in the retina turn on, as do other neurons higher up in the brain. Based on what we know, the activity of neurons in the retina is not directly associated with conscious experience of light and dark – they behave just like biological photodiodes that signal to higher centers. Somewhere in those higher centers, however, there seem to be some neurons whose activity is indeed tightly correlated with the conscious experience of light and dark. What is special about these higher neurons?

ii. Note that this information has nothing to do with how complicated the scene is, or how many different objects it appears to contain, but only with the number of alternative outcomes.

iii. This quantity is also called ^*MIB*^*complexity*, for minimum information bipartition complexity. Note that, in most cases, the bipartitions for which the normalized value of EI will be at a minimum, everything else being equal, will be bipartitions that cut the system in two halves, i.e. midpartitions [2].

iv. Complexes can also be defined using mutual information instead of effective information, by exploiting the endogenous sources of variance that may exist in an isolated system [8]. A related measure could be constructed using the formalism of ε-machines [63]. Φ would then be related to the H^μ ^of the minimal ε-machine capable of reproducing the causal structure of a process, i.e. of the ε-machine that cannot be decomposed into a collection of lower H^μ ^ε-machines.

v. An elementary description of the qualia space is given by the author in [9], chapter 13.

vi. While the entries in the matrix contain all the relevant informational relationships defining this space, they do not reveal necessarily how the space is organized in an economical and explicit manner. This may be done by employing procedures akin to multidimensional scaling although, since the matrix is asymmetrical and involves high-order terms (among subsets of elements), this may not be easy. Satisfactorily mapping the phenomenological differences between modalities, submodalities and dimensions onto the structure of qualia space will require that we thoroughly characterize and understand the latter.

vii. Of course, sensory afferents usually play a role in determining which particular conscious experience we have at any given time (they better do so, if experience is to have an adaptive relationship to the environment). Nevertheless, particular experiences can be triggered even when we are disconnected from the environment, as in dreams.

viii. Note also that a "pure" sensation of blue defines a point in this N-dimensional qualia space as much as the experience of a busy city street, full of different objects, of sounds, smells, associations, and reflections defines another point.

ix. However, certain areas such as the posterior cingulate cortex and precuneus, some lateral parietal areas, and associated paramedian thalamic nuclei, may constitute strategic crossroads for coordinating the interactions among different sensory maps and frames of reference concerning the body and the environment. Bilateral lesions to such areas may lead to a virtual breakdown of information integration in the thalamocortical system [22,24]. A global, persistent disruption of consciousness can also be produced by focal lesions of paramedian mesodiencephalic structures, which include the intralaminar thalamic nuclei. Most likely, such focal lesions are catastrophic because the strategic location and connectivity of paramedian structures ensure that distributed cortico-thalamic loops can work together as a system. 

x. Statements about the lack of direct contributions to consciousness of basal ganglia loops need to be qualified due to the difficulty of evaluating the precise effects of their selective inactivation, as well as to the unreliability of introspective assessments about the richness of one's experience, especially after brain lesions. Similar considerations apply to brain structures not discussed here, such as the claustrum, the amygdala, and the basal forebrain.

xi. A similar kind of analysis could be applied to other neurological disconnection syndromes.

xii. An explanation in terms of reduced degrees of freedom may also apply to loss of consciousness in absence and other seizures, during which neural activity is extremely high and near-synchronous over many cortical regions (Tononi, unpublished results).

xiii. While we do not yet have such a tight case for the neural correlate of blue, we are close to it with motion sensitive cells in area MT and in somatosensory cortex, at least in monkeys [64].

xv. In this sense, a particular conscious experience, its meaning, and the underlying informational relationships within a complex end up being one and the same thing. Such internalistic, relationally defined meanings generally relate to and ultimately derive from entities in the world. To the extent that the brain has a long evolutionary history and is shaped by experience, it is clear that internally specified meanings (and conscious states) bear an adaptive relationship to what is out there.
